# 18F-AlF-NOTA-octreotide PET/CT and 3D printing technology for precision diagnosis and treatment of phosphaturic mesenchymal tumors in patients with tumor-induced osteomalacia: two case reports

**DOI:** 10.3389/fendo.2024.1359975

**Published:** 2024-11-20

**Authors:** Gang Zhao, Lijuan Guan, Yongqiang Zhang, Xingzhen Shi, Wenming Luo, Maiqing Yang, Qi Wang, Zhen Liu, Yongqiang Liu, Xiaolei Ding, Jie Zhao

**Affiliations:** ^1^ Department of Orthopedics and Trauma, Weifang People’s Hospital, First Affiliated Hospital of Shandong Second Medical University, Weifang, China; ^2^ Nursing Department, Weifang Stomatology Hospital, Weifang, China; ^3^ Department of Pathology, Weifang People’s Hospital, First Affiliated Hospital of Shandong Second Medical University, Weifang, China; ^4^ College of Traditional Chinese Medicine, Changchun University of Chinese Medicine, Changchun, Jilin, China

**Keywords:** phosphaturic mesenchymal tumors, tumor-induced osteomalacia, 18F-AlF-NOTA-octreotide PET/CT imaging, 3D printing technology, case reports

## Abstract

**Objective:**

This study aims to report the application of 18F-AlF-NOTA-Octreotide PET/CT and 3D printing technology in the diagnosis and treatment of phosphaturic mesenchymal tumors (PMT) in patients with tumor-induced osteomalacia (TIO).

**Case presentation:**

A 68-year-old male patient (Case 1) was admitted to the Weifang People’s Hospital in August 2022 with complaints of “persistent pain in the bilateral flank and lumbosacral region”. 18F-AlF-NOTA-Octreotide PET/CT showed high octreotide expression in the left femoral region. A 48-year-old male patient (Case 2) was admitted to the Weifang People’s Hospital in November 2022, complaining of “pain in the lumbar region and ribs”. 18F-AlF-NOTA-Octreotide PET/CT showed high octreotide expression in the pancreatic uncinate process and the left acetabulum. They were diagnosed with hypophosphatemic osteomalacia, with a strong consideration of an underlying neuroendocrine tumor. Preoperative design of 3D virtual surgery, CAD/CAM, and 3D printing technology were used to customize the digital surgical guide plates, and the surgery was carried out. They were both finally confirmed as phosphateuric mesenchymal tumors (PMT) based on postoperative pathology and immunohistochemistry results. Both patients experienced substantial relief from their clinical manifestations after surgery.

**Conclusion:**

18F-AlF-NOTA-Octreotide PET/CT may be a precise diagnostic method for TIO, while 3D printing technology may serve as an effective and dependable adjunct for the treatment of PMT in patients with TIO.

## Introduction

Tumor-induced osteomalacia (TIO) is a rare paraneoplastic syndrome resulting from tumor-induced renal phosphate depletion and decreased bone mineralization, eventually leading to a series of adverse manifestations, including progressive skeletal pain, mobility impairments, and skeletal deformities (1, 2). TIO primarily arises due to the uncontrolled secretion of fibroblast growth factor-23 (FGF-23) by phosphaturic mesenchymal tumors (PMT) (3), preventing renal phosphate reabsorption and reducing intestinal phosphate absorption, leading to osteomalacia and the other signs and symptoms of TIO (4). The effective treatment of TIO requires thorough removal of the causative tumor using radiofrequency ablation (5, 6), and accurate localization of the tumor site is essential for successful outcomes, but it can be challenging. Previous studies have indicated that 18F-AlF-NOTA-Octreotide PET/CT can be used for the localization of neuroendocrine neoplasms (7, 8), but there is limited reporting on its application in the diagnosis and localization of TIO. 3D printing technology has been widely applied in orthopedics (9–13), but its application in TIO is also very limited. Herein, this study aims to report the application of 18F-AlF-NOTA-Octreotide PET/CT and 3D printing technology in the diagnosis and treatment of phosphaturic mesenchymal tumors (PMT) in patients with TIO.

## Case presentation

### Case 1

A 68-year-old male patient was admitted to the Weifang People’s Hospital in August 2022, complaining of “persistent pain in the bilateral flank and lumbosacral region”.

**Figure 1:**
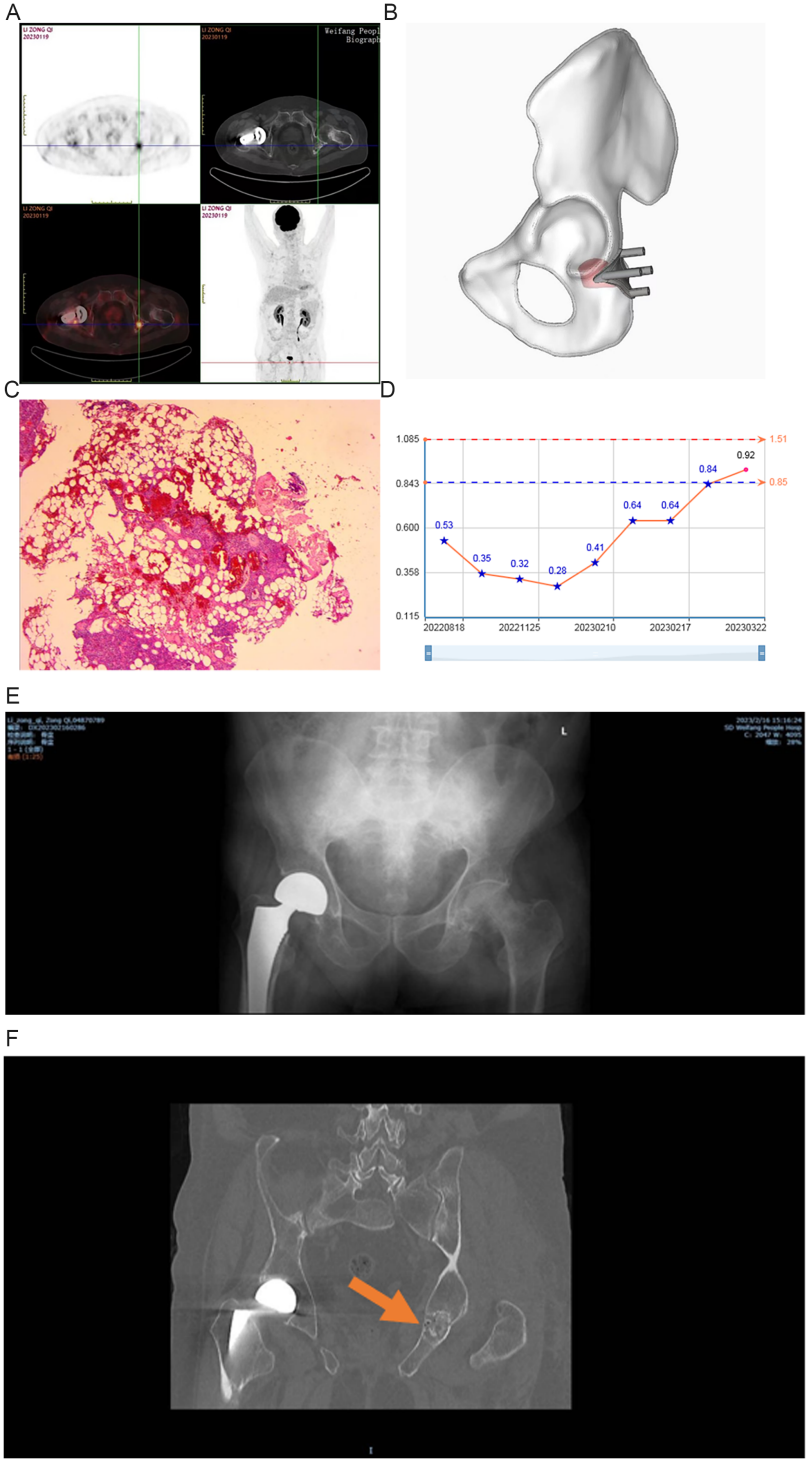
Case 1: **(A)** a: 18F-AlF-NOTA-Octreotide PET/CT imaging results; **(B)** 3D-printed guide plate; **(C)** Hematoxylin & eosin staining; **(D)** Blood phosphorus changes; **(E)** X-ray after surgery; **(F)** CT during reexamination.

The physical examination showed increased pain in the neck and shoulder area during forward flexion, extension, and neck rotation. Spurling’s test was positive, and there was tenderness over the spinous processes of the posterior neck, as well as tenderness when pressing on the inner angles of both scapulae and the trapezius muscle in the neck and shoulder area. Tenderness was evident in the thoracic spine paravertebrally and over the chest. The straight leg raise test was positive at 70° on the right side, and Patrick’s test (FABER test) was positive on the right side. The patient underwent surgical treatment for a right hip femoral neck fracture. Laboratory tests showed blood phosphorus levels of 0.53 mmol/L (normal range: 1.1-1.3 mmol/L), 25-hydroxy-VITD was 27.83 ng/ml (normal range: 8-30.5 ng/mL), and alkaline phosphatase (ALP) was 183 U/L (normal range: 40-150 U/L). 18F-AlF-NOTA-Octreotide PET/CT showed high octreotide expression in the left femoral region (**Figure 1A**). The patient was considered hypophosphatidic osteochondrosis, with a strong consideration of an underlying neuroendocrine tumor.

According to preoperative 18F-AlF-NOTA-Octreotide PET/CT imaging, preoperative design of 3D virtual surgery, CAD/CAM, and 3D printing technology were used to customize the digital surgical guide plates (**Figure 1B**). Following general anesthesia, the patient was placed in a prone position, and a Kocher-Langenbeck approach was used. The procedure involved sequential incisions through the skin, subcutaneous tissue, and deep fascia to expose the femur. A 3D-printed guide plate was then placed to determine the extent of the lesion. After examination, the guide plate showed good alignment, and Kirschner wires were drilled along the guide plate as per the direction and depth to determine the scope and cross-section of the lesion. The surface of the lesion showed a thin bone. Under the guidance of the guide plate, the grinding drill was used to remove the surface cortical bone of the lesion, exposing the localized lesion area. Soft tissue-like structures were observed within the bone, and the tumor was approximately 2 cm in size, with a grayish-red color. It had a relatively firm outer shell, while the tumor body itself was soft and elastic, with no adhesion. After complete excision and debridement of the sclerotic bone, allograft bone particles from freeze-dried cancellous bone were placed in the bone defect area, followed by layer-by-layer suturing. Blood loss was 100 mL, and the surgical time was 1 h.

The postoperative pathology showed spindle-shaped cell proliferation in the bone marrow tissue, accompanied by vascular and adipose tissue proliferation, resembling a structure similar to vascular smooth muscle lipoma. Localized chondroid calcification was also observed. Immunohistochemistry showed Vimentin (+), CD56 (+), BCL-2 (+), SATB-2 (+), CD99 (+), stat6 (cytoplasmic +), SSTR-2 (+), CD34 (vascular +), CD68 (little +), Ki-67 (3%). The patient was finally confirmed as PMT according to pathology results (**Figure 1C**).

Two months after the surgery, the patient experienced significant relief in pain in the lumbosacral and hip regions, the VAS (Visual Analog Scale) score became 0 points, and the blood phosphorus levels increased to 0.92 mmol/L. The patient was administrated with oral calcium carbonate with vitamin D3 granules until blood phosphorus levels returned to normal (**Figure 1D**). X-ray and CT after surgery indicated the local bone density was enriched after implantation (**Figures 1E, F**).

### Case 2

**Figure 2:**
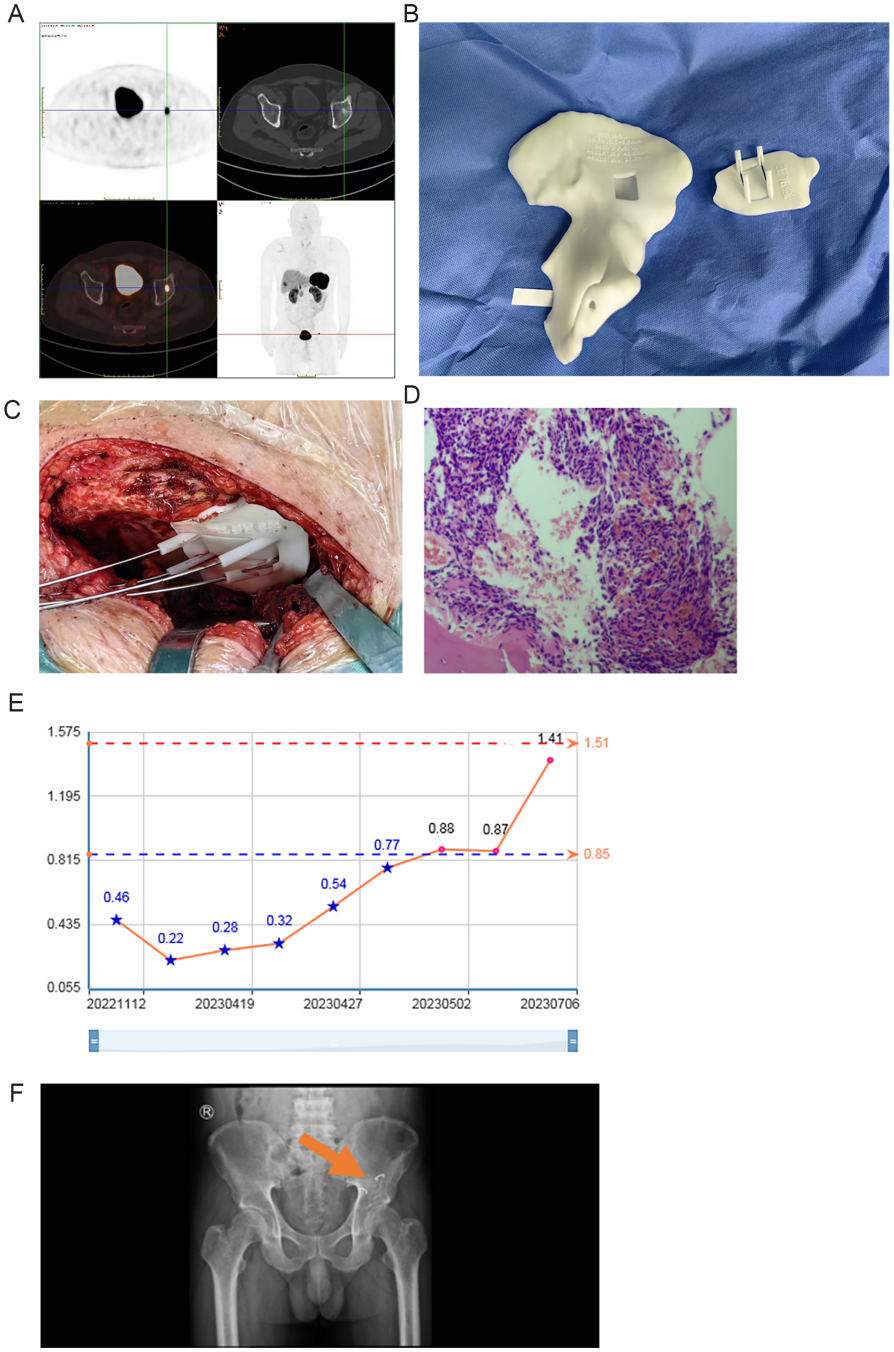
Case 2: **(A)** 18F-AlF-NOTA-Octreotide PET/CT imaging results; **(B)** 3D-printed guide plate; **(C)** 3D-printed guide plate use during surgery; **(D)** Hematoxylin & eosin staining; **(E)** Blood phosphorus changes. **(F)** X-ray after surgery.

A 48-year-old male patient was admitted to the Weifang People’s Hospital in November 2022, complaining of “pain in the lumbar region and ribs”. The physical examination showed tenderness and pain upon palpation of the thoracolumbar spinous processes, paravertebral region, and chest ribs, with discomfort during movement. Laboratory tests showed blood phosphorus at 0.46 mmol/L and the 25-hydroxy-VITD of 9.45 ng/ml. The 18F-AlF-NOTA-Octreotide PET/CT showed high octreotide expression in the pancreatic uncinate process and the left acetabulum. The patient was diagnosed with hypophosphatemic osteomalacia, with a strong consideration of an underlying neuroendocrine tumor (**Figure 2A**).

After general anesthesia, the procedure involved the use of an ilioinguinal approach, partial periosteal dissection to expose the internal plate of the ilium, and placement of a 3D-printed guide plate (**Figures 2B, C**). The examination showed that the guide plate matched the acetabulum well. Kirschner wires were drilled around the guide plate as per the preoperative design, and Kirschner wires were inserted to delineate the lesion area. Under the guidance of the guide plate, a micro saw was used to make an incision at the appropriate angle and depth in the proximal cortical bone of the lesion, which was set aside, exposing the localized lesion area. The bone knife was used to remove the lesion’s bone, which contained a small amount of soft tissue with unclear boundaries. The excision was extended beyond the preoperative targeted area until a portion of the femoral head was exposed. The deficient area was reconstructed using a bone graft from the iliac wing and compacted and rectangular bone pieces were placed, followed by fixation with arch-shaped nails. Blood loss was 200 mL, and the surgical time was 1.5 h.

The postoperative pathology results confirmed the presence of PMT (**Figure 2D**).

The patient’s systemic bone pain disappeared, and he was able to walk independently after postoperative 4 months. Blood phosphorus was 1.41 mmol/L, and the 25-hydroxy-VITD was 32.88 ng/ml in July 2023. Oral calcium carbonate D3 granules were administered postoperatively until the blood phosphorus returned to normal (**Figure 2E**). The postoperative X-ray shows an increase in local bone density after implantation (**Figure 2F**).

## Discussion

This study presents two cases of TIO due to PMT, who were examined through 18F-AlF-NOTA-Octreotide PET/CT and treated with the assistance of 3D digital surgical guide plates, which may offer valuable insights for the diagnosis and management of PMT in patients with TIO. The two patients underwent 18F-AlF-NOTA-Octreotide PET/CT because it was more accessible than 68Ga-DOTATATE PET/CT at the primary hospital where the patients were admitted. In addition, a previous study indicated that 18F-AlF-NOTA-Octreotide could achieve at least comparable performance to 68Ga-DOTATATE/NOC (14).

The diagnosis of TIO can be challenging. Computer tomography (CT), magnetic resonance imaging (MRI), and other imaging techniques might not consistently identify TIO or precisely locate lesions in unique or uncommon anatomical regions (15). In the two cases, the implementation of 18F-AlF-NOTA-Octreotide PET/CT facilitated the precise diagnosis of TIO.

3D printing technology, as a concentrated embodiment of digital technology, is an effective means to realize the individualization and precision of various orthopedic surgeries. According to a previous study exploring the role of 3D printed surgical guides in the resection and reconstruction of malignant bone tumors, the blood loss, resection length, and complication rates were significantly lower in the 3D printed surgical guide group than in the control group (16). A recent study indicated that the use of 3D-printed guides could be used to create patient-specific bone grafts to manage glenoid deformity (17). Such guides can also be used for precise bone lesion resection (18–23), allowing the precise removal of the tumors while minimally compromising the bone structure. Still, no previous study examined the use of such guides in patients with TIO. In this study, the 3D guide plate could accurately guide the direction and depth of the channel of screws and determine the cross-section, the distance, and the relationship between each other into the angle, etc., thus resulting in increased surgical accuracy and safety, shortened operating time, reduced intraoperative bleeding and side injuries. Furthermore, the application of 3D printing facilitates some parts of the operation that are more complicated and difficult in traditional surgeries. As a result, the use of 3D-printed guiding plate result in shorter surgeries and smaller blood losses (24, 25).

A limitation of the two cases reported here is that FGF23 was not measured; it is not a routine test at the authors’ hospital. Only two cases were reported here, preventing comparison with controls and the evaluation of the patient outcomes.

## Conclusion

This study indicated that 18F-AlF-NOTA-Octreotide PET/CT may be a precise diagnostic method for TIO, while 3D printing technology may serve as an effective and dependable adjunct for the treatment of TIO deprived from PMT, which provided a valuable reference for the diagnosis and treatment of TIO.

## Data Availability

The original contributions presented in the study are included in the article/supplementary material. Further inquiries can be directed to the corresponding author.
